# The Efficient Photocatalytic Degradation of Organic Pollutants on the MnFe_2_O_4_/BGA Composite under Visible Light

**DOI:** 10.3390/nano11051276

**Published:** 2021-05-13

**Authors:** Qian Li, Xiaoyu Jiang, Yongfu Lian

**Affiliations:** Key Laboratory of Functional Inorganic Material Chemistry, Ministry of Education, School of Chemistry and Materials Science, Heilongjiang University, Harbin 150080, China; 2171074@s.hlju.edu.cn (Q.L.); 2201357@s.hlju.edu.cn (X.J.)

**Keywords:** manganese ferrite, boron-doped graphene aerogel, degrading efficiency

## Abstract

The MnFe_2_O_4_/BGA (boron-doped graphene aerogel) composite was prepared by hydrothermal treatment of MnFe_2_O_4_ particles, boric acid, and graphene oxide. When applied as a photo-Fenton catalyst for the degradation of rhodamine B, the MnFe_2_O_4_/BGA composite yielded a degradation efficiency much higher than the sum of those of individual MnFe_2_O_4_ and BGA under identical experimental conditions, indicating a strong synergetic effect established between MnFe_2_O_4_ and BGA. The catalytic degradation of rhodamine B was proved to follow pseudo first-order kinetics, and the apparent reaction rate constant on the MnFe_2_O_4_/BGA composite was calculated to be three- and seven-fold that on BGA and MnFe_2_O_4_, respectively. Moreover, the MnFe_2_O_4_/BGA composite also demonstrated good reusability and could be reused for four cycles without obvious loss of photocatalytic activity.

## 1. Introduction

Recently, the spinel-type bimetal oxide MnFe_2_O_4_ nanoparticles have been intensively investigated as photo-Fenton catalysts for the degradation of organic pollutants in wastewater [1], mainly owing to their perfect chemical stability, efficient visible light response, and good catalytic performance [2,3]. However, pure MnFe_2_O_4_ does not demonstrate enough efficiency under visible light irradiation, because of its low conductivity, easy aggregation, and quick recombination of photo-generated electron-hole pairs [4]. To overcome these disadvantages, researchers have paid much attention to the composites of MnFe_2_O_4_ nanoparticles and other materials, including metal/semiconducting nanoparticles and two-dimensional sheets. Qin et al. synthesized a composite of MnFe_2_O_4_ and gold nanoparticles for photo-Fenton degradation of tetracycline (TC) under neutral pH [5]. It was found that the synergistic effect between MnFe_2_O_4_ and gold nanoparticles endowed the MnFe_2_O_4_/Au composite with quite good photo-Fenton catalytic performance. Zhao et al. prepared flower-like SnS_2--_loaded MnFe_2_O_4_ nanocomposites and demonstrated that SnS_2_ could effectively inhibit electron-hole pair recombination [6]. Later, they also reported a ternary MnFe_2_O_4_/CeO_2_/SnS_2_ photocatalyst [7], which exhibited much higher photocatalytic activity than MnFe_2_O_4_ particles toward the degradation of methylene blue (MB) under visible light irradiation. As for the two-dimensional sheets composited with MnFe_2_O_4_ nanoparticles, they are only limited to C_3_N_4_ and graphene-based materials. Vignesha et al. applied the nanocomposites (MnFe_2_O_4_/g-C_3_N_4_/TiO_2_) to the photo-degradation of methyl orange (MO) and ascribed their much-enhanced photocatalytic activity to the synergistic effect between TiO_2_, g-C_3_N_4_, and MnFe_2_O_4_ [8]. As a matter of fact, MnFe_2_O_4_ nanoparticles were commonly reported to be immobilized on graphene-based matrices to accelerate the transfer of photo-induced carriers and to reduce the electron-hole pair recombination rate. Fu et al. first reported the improved photocatalytic activity of MnFe_2_O_4_/graphene composite for the degradation of MB in the absence of hydrogen peroxide [9]. Subsequently, the MnFe_2_O_4_/graphene composite was proved to be superior to pure MnFe_2_O_4_ in the degradation of rhodamine B (RhB) [10], NH_3_ [11], MB [12], and other organic dyes [13] under visible light irradiation. Luciano et al. introduced sand into the MnFe_2_O_4_/graphene composite, which not only enhanced the photocatalytic efficiency of MB degradation under visible light irradiation, but also improved the stability and reusability of the ternary composite photocatalyst [14].

In comparison to graphene, three-dimensional (3D) graphene aerogel (GA) has the advantages of more efficient electron transfer, larger inner surface areas, and more stable skeletal structure. In recent years, the composites of iron-related photoctalysts and GA have been extensively investigated in the field of photocatalysis. In the binary Fe_2_O_3_/GA [15], Fe_3_C/GA [16], and CoFe_2_O_4_/GA [17] composites, the iron-related photoctalyst particles were observed to be uniformly dispersed in the 3D hierarchical pores of GA, which was responsible for the improved photocatalytic activity and reusability of these composites in organic pollutant degradation. In 2017, Liu et al. introduced carbon nanotubes (CNTs) to the binary α-FeOOH/GA composite and tested the photocatalytic performance of the ternary α-FeOOH/GA/CNT composite [18]. It was proved that the ternary α-FeOOH/GA/CNT composite demonstrated very good photocatalytic activity for the degradation of Orange II, RhB, MB, phenol, and bisphenol A, which could be ascribed to the efficient charge/mass transportation in the 3D porous aerogel networks. Later, Shi et al. introduced a highly hydrophilic wood-derived cellulose nanofibril (CNF) to the binary α-FeOOH/GA composite. The ternary α-FeOOH/GA/CNF composite was highly hydrophilic, which endowed this ternary composite with sufficient mass/charge transportation for the degradation of RhB, MB, MO, phenol, BPA, TC, and Cr (VI) [19].

Moreover, heteroatom doping into the 2D carbon frameworks of graphene is an efficient chemical modification technique to improve the catalytic performance of graphene-based materials, because the doped heteroatoms can tailor the surface properties of graphene, modulate the electric charge distribution of the sp^2^ hybridized carbon framework, and generate more active sites. Li et al. prepared a binary composite of CoFe_2_O_4_ particles and nitrogen-doped graphene aerogel (NGA) (CoFe_2_O_4_/NGA) and utilized it as a peroxymonosulfate activator for the degradation of benzotriazole (BTA) [20]. The degradation efficiency of BTA on the CoFe_2_O_4_/NGA composite was found to be more than double that on the CoFe_2_O_4_/GA composite, indicating that NGA was a better photocatalyst carrier than GA. It was believed that the doped N species played positive roles in the enhancement of the redox activity, electron transfer efficiency, and active site density of CoFe_2_O_4_/NGA. On the other hand, boron-doped graphene aerogel (BGA) was proved to be an effective photocatalyst for the degradation of acridine orange (AO) under visible light irradiation. The photo-degradation efficiency of AO on BGA was more than double that on undoped GA [21]. The superior photocatalytic activity of BGA compared to GA can be attributed to the more densely interconnected 3D cross-linked network structure, more internal defects, and uneven electric charge distribution in BGA, which led to the enhancement of visible light harvesting capability and a decrease in the recombination of photoinduced electron-hole pairs.

Nonetheless, to the best of our knowledge, there are still no reports on GA or nonmetal-doped GA loaded MnFe_2_O_4_ nanoparticles as photo-Fenton catalysts for the degradation of water pollutants, even though GA-loaded manganese ferrite (Mn_x_Fe_3−x_O_4_) [22] exhibited good oxygen reduction/evolution reaction activities, and the BGA-modified gas diffusion electrode was employed for the degradation of bisphenol A in a nonmetallic electrochemical advanced oxidation process [23]. In this context, a novel BGA-loaded MnFe_2_O_4_ (MnFe_2_O_4_/BGA) photo-Fenton catalyst was synthesized by hydrothermal treatment of MnFe_2_O_4_ particles, boric acid, and graphene oxide. When applied as a photo-Fenton catalyst, the MnFe_2_O_4_/BGA composite demonstrated very good catalytic performance and reusability for the degradation of organic pollutants. It is expected that the BGA-loaded MnFe_2_O_4_ would be a promising and widely applicable photo-Fenton catalyst for the effective degradation of environmental aqueous organic pollutants. 

## 2. Materials and Methods

### 2.1. Materials

Analytical grade ethanol absolute (Tianjin Zhiyuan Chemical Reagent Co., Ltd., Tianjin, China), H_3_BO_3_ (Xilong Scientific Co., Ltd., Shantou, China), FeCl_3_·6H_2_O (Shanghai Aladdin Biochemical Technology Co., Ltd., Shanghai, China), MnCl_2_·4H_2_O (Shanghai Macklin Biochemical Co., Ltd., Shanghai, China), NaOH (Sinopharm Chemical Reagent Co., Ltd., Shanghai, China), KMnO_4_ (Tianjin Kemiou Chemical Reagent Co., Ltd., Tianjin, China), H_2_SO_4_ (18 mol/L, Harbin Polytechnic Chemical Reagent Co., Ltd., Harbin, China), HCl (12 mol/L, Harbin Polytechnic Chemical Reagent Co., Harbin, China), isopropanol (IPA, Shanghai Aladdin Biochemical Technology Co., Ltd., Shanghai, China), benzoquinone (BQ, Shanghai Macklin Biochemical Co., Ltd., Shanghai, China), ammonium oxalate (AO, Shanghai Macklin Biochemical Co., Ltd., Shanghai, China), H_2_O_2_ (30 wt%, Sinopharm Chemical Reagent Co., Ltd., Shanghai, China), rhodamine B (RhB, Tianjin Kemiou Chemical Reagent Co., Ltd., Tianjin, China), methylene blue (MB, Damao Chemical Reagent Factory, Tianjin, China), crystal violet (Shanghai Aladdin Biochemical Technology Co., Ltd., Shanghai, China), specpure graphite powder (1000 mesh, Shanghai Xili Carbon Co. Ltd., Shanghai, China) and deionized water were used throughout the experiments. 

### 2.2. Preparation of the MnFe_2_O_4_/BGA Composite

The MnFe_2_O_4_ nanoparticles were prepared by a co-precipitation procedure [24]. Briefly, MnCl_2_·4H_2_O (0.3919 g) and FeCl_3_·6H_2_O (1.0931 g) were dissolved in deionized water (50 mL), and then adjusted with 6 mol/L NaOH until pH = 12 under vigorous mechanical stirring. MnFe_2_O_4_ nanoparticles precipitated while the above suspensions were stirred at 100 °C for 4 h. After washing alternately with water and ethanol 3 times to remove some adsorbed impurities and drying in a vacuum oven at 60 °C for 12 h, the product was ground to fine powder in a mortar.

Graphene oxide (GO) was prepared from specpure graphite powder by a modified Hummers method [25]. In brief, KMnO_4_ (3.0 g) was gradually added to a mixture of concentrated H_2_SO_4_ (23 mL) and graphite powder (1.0 g) cooled in an ice bath (to keep the temperature in the range of 0–10 °C), and then magnetically stirred for 0.5 h at 50 °C. When 18 mL of deionized water was slowly added, the mixture was stirred for another 10 min at 95 °C. After 35 mL deionized water and 3 mL H_2_O_2_ (30 wt%) were successively added, the reactant mixture was centrifuged (DT5A, Hunan Kaida Scientific Instruments Co., Ltd., Changsha, China) at 5000 rpm for 10 min. The collected centrifugal precipitate was dispersed in 100 mL 10% HCl and then centrifuged again. After the above dispersion–centrifugation process was repeated 3 times, the centrifugal precipitate was collected and dispersed in 100 mL deionized water and subjected to the recycle of dispersion–centrifugation process until pH = 6.0. The exfoliated GO was obtained after the centrifugal precipitate was dried at 50 °C for 24 h under vacuum.

For the synthesis of MnFe_2_O_4_/BGA composite, MnFe_2_O_4_ (65 mg) and H_3_BO_3_ (230 mg) were added into 25 mL aqueous dispersion of GO (2 mg/mL). After stirring for 30 min at room temperature, the mixture was transferred to a Teflon-lined stainless steel autoclave and hydrothermally treated at 180 °C for 12 h. When the autoclave cooled down to ambient temperature, the product was collected and soaked in an appropriate amount of aqueous solution of ethanol (20%) for 12 h. Finally, the MnFe_2_O_4_/BGA composite was obtained after freeze-drying treatment of the product for 48 h. Analogously, BGA and the MnFe_2_O_4_/GA composite were also synthesized in the same procedure without the addition of MnFe_2_O_4_ or H_3_BO_3_, respectively. 

### 2.3. Photo-Fenton Degradation of Organic Pollutants on the MnFe_2_O_4_/BGA Composite

The photo-Fenton activity of MnFe_2_O_4_/BGA composite was evaluated by photocatalytic degradation of such organic dyes as RhB, crystal violet, or MB under visible-light irradiation. Typically, 2 mg catalyst was dispersed in 50 mL aqueous solution of organic dye (10 mg/L) and stirred for 90 min in the dark. A 300 W xenon lamp (CEL-HXF-300, Beijing, China) coupled with a 400 nm cut-off filter was used as a visible light source. The photo-Fenton degradation was initiated by injection of 1.0 mL 30% H_2_O_2_ under visible light. At certain time intervals, 3 mL aliquots were withdrawn by pipette and filtered with 0.22 μm membrane, and the concentration of residual organic dyes in the filtrates was detected by their UV-vis spectra.

### 2.4. Characterization Methods

Morphologies were observed by scanning electron microscopy (SEM, QUANTA 200S, FEI, Eindhoven, The Netherlands) and a high-resolution transmission electron microscope (TEM, JEM2100, JEOL, Tokyo, Japan). Structure and composition were investigated by X-ray diffraction (XRD, D8 Advance, Bruker, Berlin, Germany) and X-ray photoelectron spectroscopy (XPS, KRATOS, Stretford, UK). Brunauer-Emmett-Teller (BET) surface area was collected by N_2_ adsorption–desorption method at 77 K on a Micromeritics ASAP 2010M analyzer. Fourier transform infrared spectra (FTIR) were recorded by a Perkin Elmer (Waltham, MA, USA) Spectrum 100FT-IR spectrometer. The concentration of organic dyes was measured by UV-vis absorption spectrophotometer (UV-3600, Shimadzu, Kyoto, Japan). Thermal stability of the sample was estimated using thermo-gravimetric analysis (TGA, SDT2960). Raman scattering was carried out on a Jobin Yvon (Palaiseau, France) HR 800 micro-Raman spectrometer with 458 nm excitation from a 20 mW air-cooled argon ion laser. The magnetic behavior of MnFe_2_O_4_/BGA was recorded with an MPMS-3 superconducting quantum interference device (SQUID) magnetometer in the applied field range of ±20 kOe at room temperature. The UV–vis diffuse reflectance spectra (DRS) of the samples were determined using a UV–vis spectrophotometer combined with the powerful operating software UVProbe (Shimadzu/UV-2550). Cyclic voltammetry (CV) and electrochemical impedance spectroscopy (EIS) were conducted on an SP-300 electrochemical workstation (Bio-Logic, Seyssinet-Pariset, France) in a three-electrode system with 1 mol/L Na_2_SO_4_ as electrolyte, and the working, reference, and counter electrodes were MnFe_2_O_4_ (BGA, MnFe_2_O_4_/GA or MnFe_2_O_4_/BGA), Ag/AgCl electrode, and Pt foil, respectively. Electron spin resonance (ESR) spectra were recorded on a Bruker A300 ESR spectrometer. The recombination rate of electron-hole pairs was detected with the Edinburgh FLS1000 fluorescence and phosphorescence spectrometer.

## 3. Results

### 3.1. Materials Characterizations

**Figure 1:**
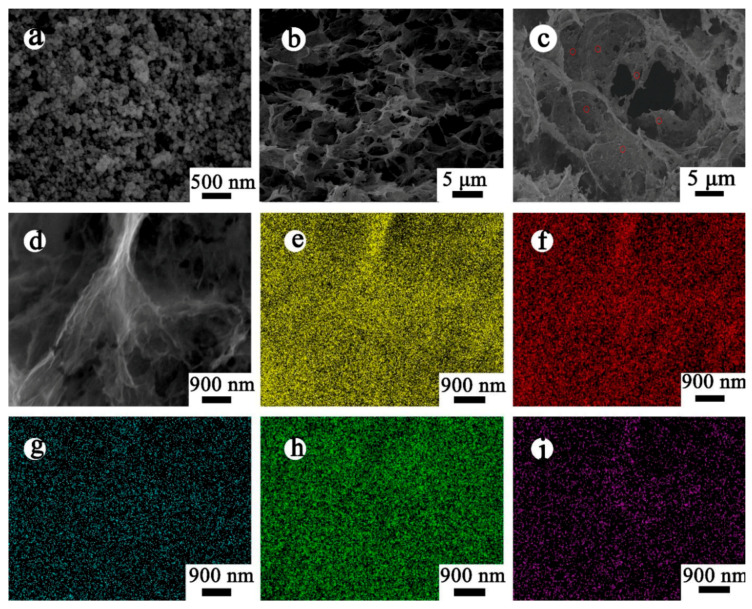
SEM images of (**a**) pure MnFe_2_O_4_, (**b**) BGA, and (**c**) MnFe_2_O_4_/BGA composite; (**d**) the elemental mapping region and (**e**–**i**) corresponding elemental mapping (C, O, Mn, Fe and B) images in MnFe_2_O_4_/BGA.

The morphology of MnFe_2_O_4_/BGA composite was explored through SEM and TEM observations. From Figure 1a,b, it can be seen that the as-prepared MnFe_2_O_4_ is composed of irregular particles with an average size around 60 nm, and the bare BGA forms a well-defined and interconnected 3D porous network structure. The average pore size is around 4 micrometers, and the pore walls consist of thin layers of stacked graphene nanosheets. Figure 1c shows that a large number of MnFe_2_O_4_ nanoparticles are evenly anchored on the 3D hierarchical networks of BGA. SEM mapping technology was also applied to the analysis of element distribution. As demonstrated in Figure 1e–i, C, O, Mn, Fe, and B elements are uniformly distributed in the MnFe_2_O_4_/BGA composite, among which Fe, Mn and O are mainly sited on the MnFe_2_O_4_ nanocrystals, while B and C are on the graphene sheets owing to the doping of B into the graphene layers. Therefore, it was concluded that the MnFe_2_O_4_ nanoparticles are homogeneously loaded on the inner surface of BGA, and that the agglomeration of MnFe_2_O_4_ is significantly suppressed, which would facilitate the increase in specific surface and the access of organic pollutants to photo-Fenton catalyst.

**Figure 2:**
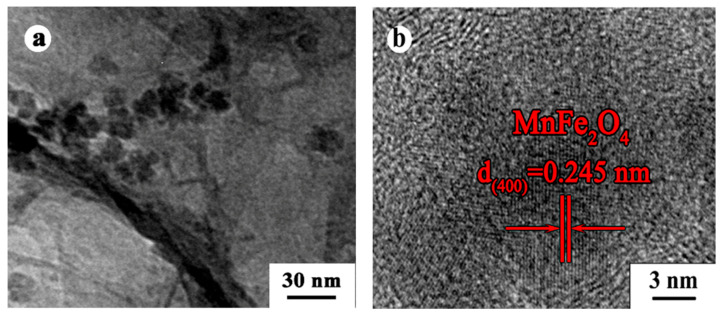
(**a**) TEM and (**b**) HRTEM images of the MnFe_2_O_4_/BGA composite.

Transmission electron microscope observation also confirmed the formation of MnFe_2_O_4_/BGA composite. From Figure 2a, it can be seen that the MnFe_2_O_4_ nanoparticles with an average size around 16 nm are randomly distributed in the hierarchical networks of BGA. From the HRTEM image shown in Figure 2b, a MnFe_2_O_4_ nanoparticle is observed. It is obvious that the typical d-spacing of 0.245 nm for those well-resolved lattice fringes corresponds to the (400) plane of MnFe_2_O_4_ crystal.

**Figure 3:**
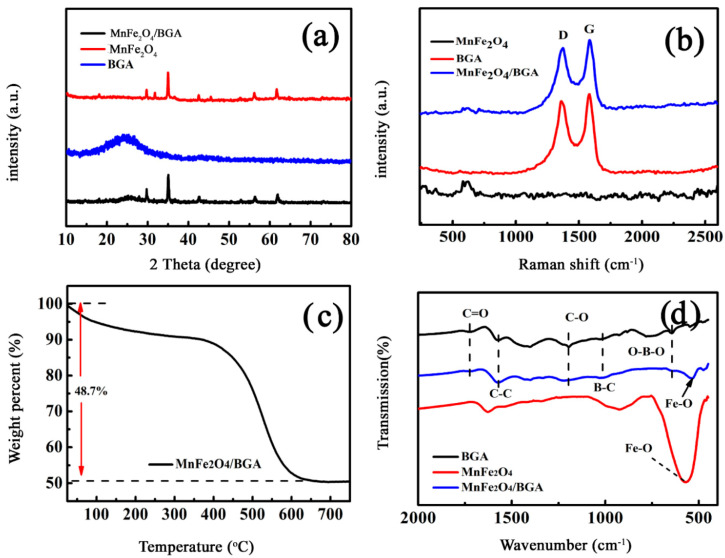
(**a**) XRD patterns, (**b**) Raman spectra, (**c**) TGA of the MnFe_2_O_4_/BGA composite (air flow at a heating rate of 10 °C/min), and (**d**) FT−IR spectra of BGA, MnFe_2_O_4_, and MnFe_2_O_4_/BGA.

Shown in Figure 3a are the XRD patterns of BGA, MnFe_2_O_4_, and MnFe_2_O_4_/BGA composite. The detected diffraction peaks could be ascribed to the crystal planes of cubic spinel structural MnFe_2_O_4_ by referring to JCPDS 10-0319 [26], except for the broad one at 24.4° ascribable to the (002) crystal plane of graphene. In line with the Debye Scherrer equation, the average crystallite sizes of MnFe_2_O_4_ nanoparticles in MnFe_2_O_4_ and MnFe_2_O_4_/BGA composite are 60 and 20 nm, respectively, which are consistent with those observed in Figure 1a or Figure 2a. Thus, the introduction of BGA could effectively restrain the aggregation of MnFe_2_O_4_ nanoparticles, while the characteristic structures of MnFe_2_O_4_ and BGA were retained in MnFe_2_O_4_/BGA composite during hydrothermal treatment. The large decrease in the average size of MnFe_2_O_4_ nanoparticles after hydrothermal processing is beneficial both for their uniform distribution in the 3D porous networks of BGA and for the increase in the specific areas of MnFe_2_O_4_/BGA composite.

Displayed in Figure 3b are the Raman spectra of MnFe_2_O_4_, BGA, and MnFe_2_O_4_/BGA composite. The characteristic D and G scattering bands of graphite are clearly observed for BGA and MnFe_2_O_4_/BGA composite. It is worth noting that the G-band shifts from 1599 cm^−1^ for GO to around 1584.5 cm^−1^ for BGA and MnFe_2_O_4_/BGA composite after hydrothermal processing, evidencing that GO was largely reduced. Furthermore, the D band of BGA or MnFe_2_O_4_/BGA is wider than that of GA, confirming the successful incorporation of B atoms into the carbon matrix of graphene [21]. In addition, the intensity ratios between D and G bands (I_D_/I_G_) are nearly same for BGA and MnFe_2_O_4_/BGA composite, implying that the loading of MnFe_2_O_4_ nanoparticles has little effect on the porous network structure of BGA. On the other hand, the characteristic scattering peak of MnFe_2_O_4_ is also observable at 612 cm^−1^ in MnFe_2_O_4_ and MnFe_2_O_4_/BGA, offering additional evidence for the formation of MnFe_2_O_4_/BGA composite. 

The thermo-stability of MnFe_2_O_4_/BGA composite was estimated by thermogravimetric analysis (TGA). It can be observed from Figure 3c that the greatest weight loss occurs in the temperature range of 400–600 °C, corresponding to the burning of the carbon skeletons of BGA. On the other hand, the weight ratio of MnFe_2_O_4_ to GO deduced from the TGA curve is consistent with the mixing weight ratio of MnFe_2_O_4_ to GO before hydrothermal processing, indicating that the doped boron element in BGA is not high enough to be detectable by TGA. 

Figure 3d displays the FT-IR spectra of as-synthesized MnFe_2_O_4_, BGA, and MnFe_2_O_4_/BGA composite. It is clear that the bands ascribed to the sp^2^-hybridized C=C in-plane, the B-C, and O-B-O bond stretching vibrations are observed at 1573, 1030, and 655 cm^−1^, respectively [27,28,29], indicative of the successful doping of boron in the graphene frameworks both in BGA and in MnFe_2_O_4_/BGA composite [30]. The peak at about 1721 cm^−1^ is due to the C=O stretching vibrations from carbonyl. Additionally, the peak at 1195 cm^−1^ is attributed to the infrared vibration of the C-O bond. Obviously, the distribution of unreduced oxygen-containing groups on BGA facilitates the loading of MnFe_2_O_4_ nanoparticles. Furthermore, the characteristic intense peak observed at 571 cm^−1^ is due to the lattice absorption of Fe-O in MnFe_2_O_4_, which shifted to 541 cm^−1^ in the MnFe_2_O_4_/BGA composite [31,32]. The shifting in the Fe-O bond vibrations is indicative of the presence of a relatively strong interaction between MnFe_2_O_4_ and BGA in the MnFe_2_O_4_/BGA composite, which might be a result of the competitive effect between the electron-deficient boron element and the positively charged iron ions toward the valence electrons of oxygen atoms in the lattice of MnFe_2_O_4_ nanoparticles [33].

**Figure 4:**
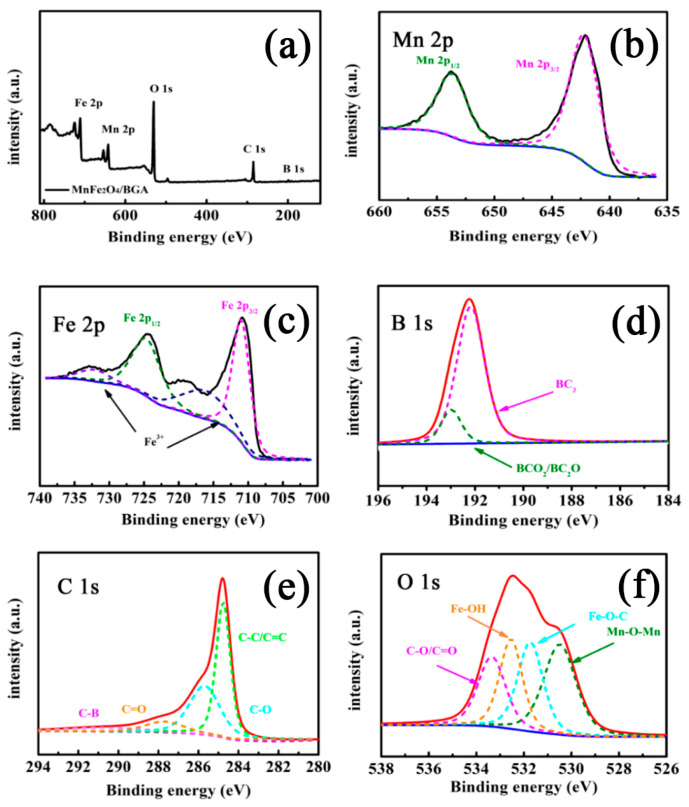
The (**a**) survey and deconvoluted (**b**) Mn 2p, (**c**) Fe 2p, (**d**) B 1s, (**e**) C 1s as well as (**f**) O 1s XPS spectra of the MnFe_2_O_4_/BGA composite.

XPS analyses were employed to elucidate the surface chemical bonding states of the MnFe_2_O_4_/BGA composite. As shown in Figure 4a, the survey spectrum evidences the existence of Mn 2p, Fe 2p, C 1s, O 1s, and B 1s regions. It is should be noted that the B 1s peak locates at 192.5 eV, which is larger than that of elemental B (187.1 eV), indicating that B atoms from H_3_BO_3_ are successfully doped into the carbon networks of graphene after hydrothermal processing [23]. Based on the XPS quantification analysis, the molar content of boron in the MnFe_2_O_4_/BGA composite was determined to be 0.93%. In the deconvoluted Mn 2p spectrum (Figure 4b), the two peaks appearing at binding energies of 642.2 and 653.6 eV are attributed to the Mn 2p_3/2_ and Mn 2p_1/2_, respectively, confirming the presence of Mn^2+^ ions in the MnFe_2_O_4_/BGA composite. In the deconvoluted Fe 2p spectrum (Figure 4c), the two main peaks at 710.9 and 724.6 eV are assigned to the Fe 2p_3/2_ and Fe 2p_1/2_, respectively, and the two satellite peaks detected at 716.2 and 732.1 eV offer further support for the presence of the Fe^3+^ ions in the MnFe_2_O_4_/BGA composite [34,35]. In the deconvoluted B 1s spectrum (Figure 4d), the intense peak at 192.2 eV corresponds to in-plane -BC_3_ type bond, which was formed by the substitution of B for C in the hexagonal lattice of graphene [21]. However, the weak peak at 192.98 eV was ascribed to the presence of boric acid ester (-BC_2_O) and boronic acid (-BCO_2_) moieties [23]. Therefore, the deconvoluted B 1s spectrum confirms the successful doping of B atoms in the skeleton of graphene during hydrothermal processing. The incorporation of the B atom into the carbon skeleton can alter the original sp^2^-hybridized structure, induce uneven charge distribution, and form new active regions in favor of activating reactions. Particularly, the B atom with positive charge polarization substitution position (-BC_3_) was the dominant species, which could serve as the activation region to migrate electrons rapidly, and thereby can facilitate the efficient separation of photogenerated carriers. In the deconvoluted C 1s spectrum (Figure 4e), the peak at 284.7 eV is ascribed to the sp^2^ structure of C-C/C=C in graphene, and the peaks at 282.3, 285.6, and 287.7 eV could be assigned to C-B, C-O, and C=O, respectively [36]. In the deconvoluted O 1s spectrum (Figure 4f), the four fitted peaks centered at 530.5, 531.7, 532.5, and 533.3 eV are assigned to the Mn-O-Mn, Fe-O-C, Fe-OH, and C-O/C=O bands, respectively. Notably, the presence of Fe-O-C illustrates the strong interaction between the iron species and graphene, which is beneficial for the rapid transfer of electrons during the photo-Fenton reaction. In addition, both the deconvoluted C 1s and O 1s spectra evidence that the MnFe_2_O_4_/BGA composite is rich in hydroxyl, carbonyl, and carboxylic acid functionalities on its surface, which is consistent with the results of its FT-IR spectrum (Figure 3d).

**Figure 5:**
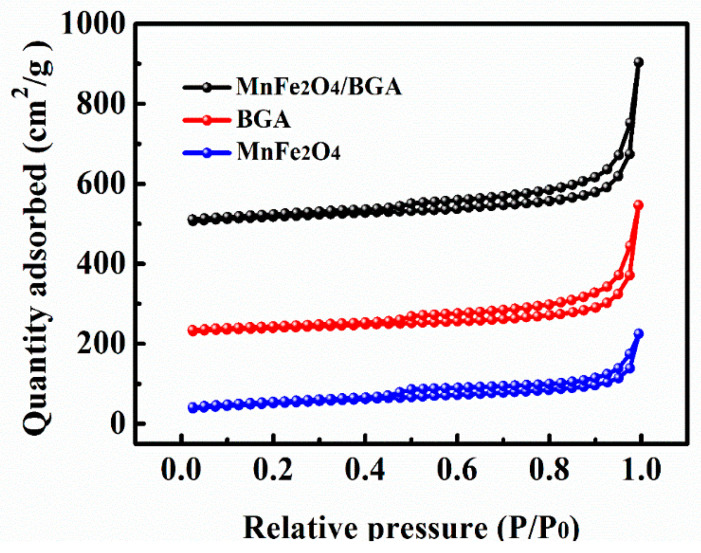
N_2_ adsorption/desorption isotherms of MnFe_2_O_4_, BGA, and MnFe_2_O_4_/BGA.

Displayed in Figure 5 are the N_2_ adsorption–desorption isotherms of MnFe_2_O_4_, BGA, and MnFe_2_O_4_/BGA. It can be seen that all of them belong to the typical type IV isotherm with an H3 hysteresis loop between the adsorption and desorption curves at higher relative pressure, indicating that these samples were composed of either flaky granular or fractured pore materials with flat slit-, crack-, or wedge-shaped mesoporous structures [37]. Moreover, the calculated specific surface area of MnFe_2_O_4_/BGA (136.7 m^2^/g) is much larger than that of BGA (108.2 m^2^/g) or pure MnFe_2_O_4_ (57.9 m^2^/g). Therefore, the MnFe_2_O_4_/BGA composite might offer more reactive sites to accelerate the generation of free radicals.

**Figure 6:**
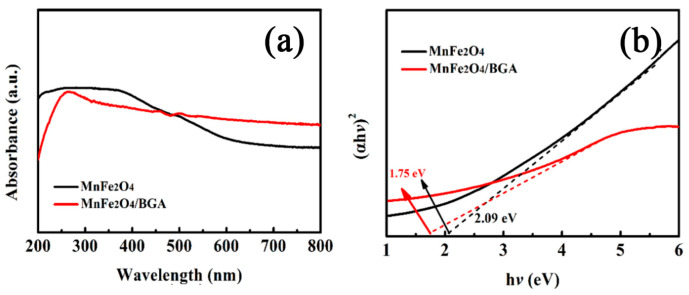
(**a**) UV–Vis DRS spectra and (**b**) the (*αhν*)^2^ versus *hν* curves of MnFe_2_O_4_ and MnFe_2_O_4_/BGA.

Shown in Figure 6a are the UV–vis DRS spectra of pure MnFe_2_O_4_ and MnFe_2_O_4_/BGA, respectively. It is obvious that the MnFe_2_O_4_/BGA composite exhibits a much higher absorption band than pure MnFe_2_O_4_ in the visible and near-IR regions, evidencing its enhanced absorption property for visible and near-IR light. Furthermore, in line with Figure 6b, the optical band gap of MnFe_2_O_4_/BGA composite is estimated to be 1.75 eV, which is smaller than that of pure MnFe_2_O_4_ (2.09 eV). Thus, with the combination of BGA, the energy gap of MnFe_2_O_4_ in the MnFe_2_O_4_/BGA composite narrows, which is beneficial to the generation and separation of the photo-induced electron-hole pairs, and eventually to the improvement in photocatalytic performance under visible light irradiation.

### 3.2. Degradation of Organic Pollutants on the MnFe_2_O_4_/BGA Composite 

#### 3.2.1. The Optimized Experimental Conditions of Photo-Fenton Catalytic Degradation

**Figure 7:**
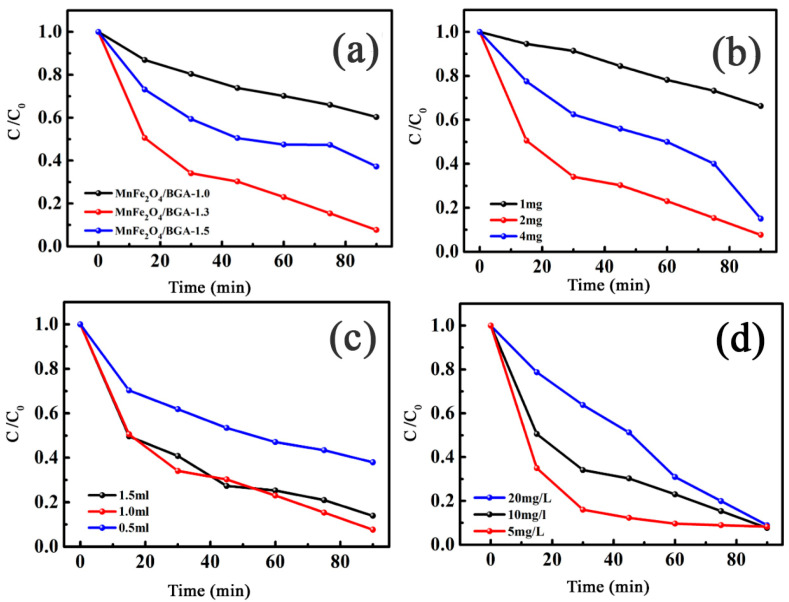
Effects of experimental parameters on degradation of RhB: (**a**) composition of MnFe_2_O_4_/BGA (dose of catalyst: 2 mg, volume of 30% H_2_O_2_: 1.0 mL, [RhB]: 10 mg/L, volume of RhB solution: 50 mL), (**b**) catalyst dosage (MnFe_2_O_4_/BGA−1.3, volume of 30% H_2_O_2_: 1.0 mL, [RhB]: 10 mg/L, volume of RhB solution: 50 mL), (**c**) volume of 30% H_2_O_2_ (MnFe_2_O_4_/BGA−1.3, catalyst dose: 2 mg, [RhB]: 10 mg/L, volume of RhB solution: 50 mL), and (**d**) initial RhB concentration (MnFe_2_O_4_/BGA−1.3, catalyst dose: 2 mg, volume of 30% H_2_O_2_: 1.0 mL, volume of RhB solution: 50 mL).

For the optimization of photo-Fenton experimental conditions, the catalytic degradation of MnFe_2_O_4_/BGA composites on RhB was investigated under visible light irradiation. Shown in Figure 7a is the degradation efficiency dependence on the mass ratio of MnFe_2_O_4_ to BGA in MnFe_2_O_4_/BGA composite. With the increase in the mass ratio from 1.0 to 1.3, the degradation efficiency was greatly improved, from 39.6% to 92.3%. However, with the further increase in the mass ratio from 1.3 to 1.5, the degradation efficiency was largely reduced to 62.7%. The influence of the dosage of MnFe_2_O_4_/BGA composite on the degradation efficiency of RhB is shown Figure 7b. With the increase in the catalyst dosage from 1 to 2 mg, the degradation efficiency increased from 33.7% to 92.3% in 90 min. Nevertheless, with a further increase in the catalyst dosage from 2 to 4 mg, the degradation efficiency slightly declined to 85%. Shown in Figure 7c is the degradation efficiency dependence on the dosage of H_2_O_2_. With the increase in the dosage of H_2_O_2_ from 0.5 to 1.0 mL, the degradation efficiency increased rapidly from 62% to 92.3%. However, with the further increase of H_2_O_2_ from 1.0 mL to 1.5 mL, the degradation efficiency decreased to 86.1%. The effect of RhB initial concentration on its photo-degradation on the MnFe_2_O_4_/BGA composite was also examined. As demonstrated in Figure 7d, the degradation efficiency within 75 min was monotonically decreased with the increase in the initial RhB concentration from 5 to 10 and to 20 mg/L. Nonetheless, the differences in degradation efficiency among the three cases became smaller and smaller after 45 min, and the degradation efficiency ultimately reached 91.7%, 92.3%, and 91.1%, respectively, at 90 min. Therefore, the optimized parameters in photo-Fenton catalytic degradation of RhB on MnFe_2_O_4_/BGA composite were summarized as follows: 2 mg MnFe_2_O_4_/BGA−1.3, 1.0 mL H_2_O_2_, 10 mg/L RhB in 50 mL aqueous solution.

#### 3.2.2. Degradation of Rhodamine B on Relevant Photo-Fenton Catalysts

**Figure 8:**
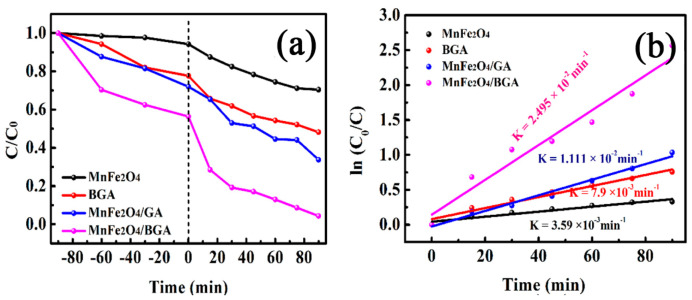
(**a**) The photo-Fenton degradation and (**b**) corresponding pseudo first-order kinetic curves of RhB on different catalysts in the presence of H_2_O_2_ under visible light (catalyst dose: 2 mg, volume of 30% H_2_O_2_: 1.0 mL, [RhB]: 10 mg/L, volume of RhB solution: 50 mL). The negative minutes of the x−axis shown in Figure 8a stand for the period of pre-adsorption in the dark without addition of H_2_O_2_.

For the comparison of degradation efficiency of relevant photo-Fenton catalysts, the degradation of RhB was investigated under the above optimized conditions on MnFe_2_O_4_, BGA, MnFe_2_O_4_/GA, and MnFe_2_O_4_/BGA, respectively. It can be seen from Figure 8a that the degradation efficiency of MnFe_2_O_4_/BGA to RhB is much larger than that of MnFe_2_O_4_/GA, indicative of the importance of boron doping in GA. Moreover, the degradation efficiency of RhB within 90 min was 28.1%, 53.1%, and 92.3% on MnFe_2_O_4_, BGA, and MnFe_2_O_4_/BGA, respectively. Because the degradation efficiency on MnFe_2_O_4_/BGA composite is much larger than the sum of the degradation efficiencies on individual MnFe_2_O_4_ and BGA under identical experimental conditions, it was concluded that a synergistic effect must be established between MnFe_2_O_4_ and BGA during the photo-Fenton degradation of RhB. It could be discovered from Figure 8b that the catalytic degradation of RhB by MnFe_2_O_4_, BGA, MnFe_2_O_4_/GA, and MnFe_2_O_4_/BGA follows the pseudo first-order kinetics, and the calculated reaction rate constants are 3.59 × 10^−3^, 7.9 × 10^−3^, 1.11 × 10^−2^, and 2.495 × 10^−2^ min^−1^, respectively. In line with the definition of synergistic index (SI) [38], SI was calculated to be 2.16, i.e., the synergistic effect yielded an additional 116% efficiency to the degradation of RhB. Thus, it was concluded that the photocatalytic potency of MnFe_2_O_4_/BGA composite is much higher than pure MnFe_2_O_4_, pure BGA, and MnFe_2_O_4_/GA composite.

**Figure 9:**
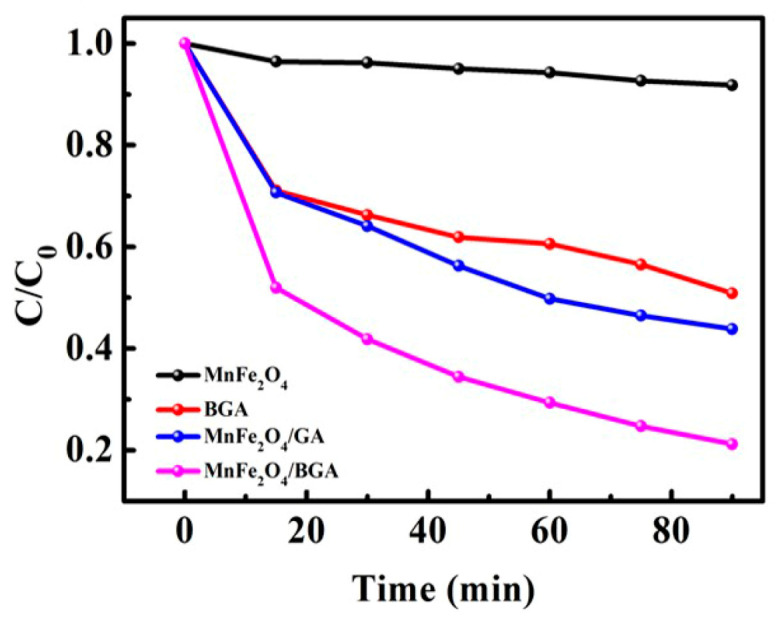
The degradation of RhB on different catalysts in the presence of H_2_O_2_ in dark (catalyst dose: 2 mg, volume of 30% H_2_O_2_: 1.0 mL, [RhB]: 10 mg/L, volume of RhB solution: 50 mL).

In order to compare with the photo-Fenton degradation, the degradation of RhB in the dark using various photocatalysts and H_2_O_2_ was also carried out, and the results are displayed in Figure 9. It can be seen from Figure 9 that the degradation rates of RhB are 8.3%, 49.2%, 56.16%, and 78.8% for MnFe_2_O_4_, BGA, MnFe_2_O_4_/GA, and MnFe_2_O_4_/BGA, respectively, which are uniformly lower than observed with the corresponding photo-Fenton degradation processes. Nonetheless, a synergistic effect was also observed between MnFe_2_O_4_ and BGA during the Fenton degradation of RhB.

#### 3.2.3. Degradation of Crystal Violet and Methylene Blue on the MnFe_2_O_4_/BGA Composite 

**Figure 10:**
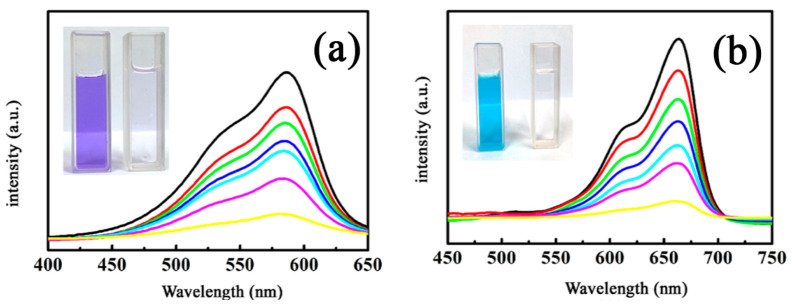
Absorption spectra of (**a**) crystal violet and (**b**) methylene blue solutions in the presence of the MnFe_2_O_4_/BGA composite for the time interval of 15 min; insets are pictures taken at 0 and 90 min, respectively (MnFe_2_O_4_/BGA−1.3; catalyst dose: 2 mg, volume of 30% H_2_O_2_: 1.0 mL, [dye]: 10 mg/L, volume of dye solution: 50 mL).

The broad application of as-synthesized MnFe_2_O_4_/BGA composite was studied for degradation of organic pollutants. The organic pollutants applied in textile industry, including crystal violet (C_25_H_30_N_3_Cl) and methylene blue (C_16_H_18_N_3_ClS), were selected as degradation targets. All are listed as carcinogenic substances with molecular structures containing multiple benzene rings. Shown in Figure 10 are the UV-vis spectra of the organic pollutants in aqueous solutions recorded after catalytic degradation on the MnFe_2_O_4_/BGA composite at certain reaction intervals. It is obvious that the absorbance for the typical absorption peaks of the organic pollutants decreases gradually with prolonged time, and all of the solutions become colorless at 90 min, as displayed in the insets of Figure 10. Thus, the Fenton-like process on the MnFe_2_O_4_/BGA composite is non-selective for the degradation of these organic pollutants, indicative of the wide applicability of the MnFe_2_O_4_/BGA composite.

**Figure 11:**
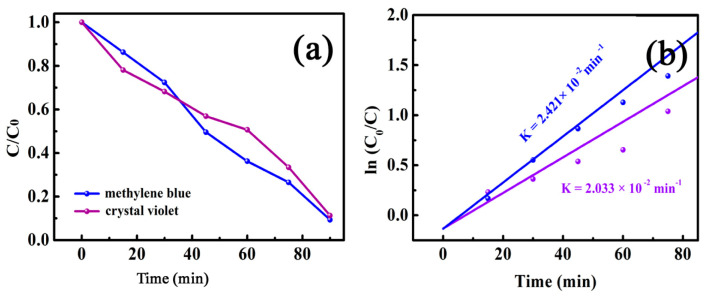
(**a**) The photo-Fenton degradation and (**b**) corresponding pseudo first-order kinetic curves of crystal violet and methylene blue solutions on MnFe_2_O_4_/BGA−1.3 in the presence of H_2_O_2_ under visible light (catalyst dose: 2 mg, volume of 30% H_2_O_2_: 1.0 mL, [dye]: 10 mg/L, volume of dye solution: 50 mL).

Displayed in Figure 11 are the catalytic degradation performance curves of MB and crystal violet on the MnFe_2_O_4_/BGA composite. Nonetheless, the degradation efficiency of MB and crystal violet reached 90.6% and 88.7% within 90 min, respectively, confirming the wide applicability of the MnFe_2_O_4_/BGA composite for these organic pollutants. Moreover, as shown in Figure 11b, these organic pollutant degradation kinetics based on pseudo first-order fit well with the experimental data under visible light irradiation. The apparent reaction rate constants were calculated to be 2.421 × 10^−2^ and 2.033 × 10^−2^ min^−1^, when MB and crystal violet were photo-Fenton degraded on the MnFe_2_O_4_/BGA composite, respectively, which further supports the very good photocatalytic performance of the MnFe_2_O_4_/BGA composite for photodegradation of these organic dyes under visible light irradiation. In comparison to the data reported previously, the calculated reaction rate constant of MnFe_2_O_4_/BGA toward MB is 4.6 times that of MnFe_2_O_4_/rGO (5.26 × 10^−3^ min^−1^) [12] and 1.7 times that of 10% MnFe_2_O_4_/rGO (1.443 × 10^−2^ min^−1^) [39], evidencing the much improved photo-Fenton degradation activity of the MnFe_2_O_4_/BGA composite.

#### 3.2.4. Stability and Reusability of the MnFe_2_O_4_/BGA Composite 

**Figure 12:**
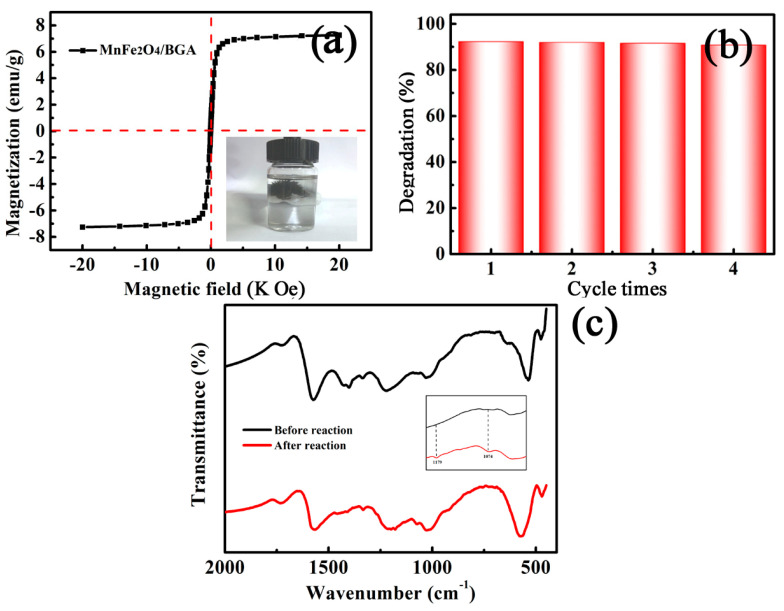
(**a**) The magnetic hysteresis loop of MnFe_2_O_4_/BGA and picture of MnFe_2_O_4_/BGA adsorbed by a magnet; (**b**) Reusability of the MnFe_2_O_4_/BGA composite in 4 successive cycles for the degradation of RhB (MnFe_2_O_4_/BGA−1.3; catalyst dose: 2 mg, volume of 30% H_2_O_2_: 1.0 mL, [RhB]: 10 mg/L, volume of RhB solution: 50 mL), and (**c**) FT−IR spectra of fresh MnFe_2_O_4_/BGA (in black) and recovered MnFe_2_O_4_/BGA (in red).

Effective magnetic separation of the MnFe_2_O_4_/BGA nanocomposite is important for its recyclable use in wastewater treatment. As shown in Figure 12a, the MnFe_2_O_4_/BGA composite displays a symmetrical S-shaped magnetization curve, evidencing the superparamagnetic behavior of this composite. The saturation magnetization was measured to be 7.25 emu g^−1^, which is high enough for the magnetic separation of the catalyst with an external magnet in the degradation solution of RhB (see the inset of Figure 12a).

Undoubtedly, the stability of the catalyst is quite crucial for practical applications. Batch experiments were carried out to evaluate the photocatalytic stability of the MnFe_2_O_4_/BGA composite for RhB degradation. It can be seen from Figure 12b that no apparent deactivation was observed after 4 cycles, indicative of the high stability and repeatability of the MnFe_2_O_4_/BGA composite for visible-light driven photocatalytic degradation of RhB. In line with the definition of “turnover number” proposed by Gomathi Devi and Shyamala [40], the turnover number after the 4th cycle was calculated to be 0.2611. Moreover, the recovered MnFe_2_O_4_/BGA composite (after the 4th cycle) was investigated by FT-IR spectroscopy. Shown in Figure 12c are the FT-IR spectra of the fresh and recovered MnFe_2_O_4_/BGA composite. It is obvious that the characteristic IR absorption peaks of the initial MnFe_2_O_4_/BGA composite observed in Figure 3d are still retained in the recovered sample. Nonetheless, two weak bands detected at 1074 and 1179 cm^−1^ in the recovered sample (see the inset of Figure 12c) were assignable to the bending vibration of aromatic C-H bonds in an intermediate product formed during the degradation process. Meanwhile, the lattice absorption of Fe-O detected in the recovered MnFe_2_O_4_/BGA composite was blue-shifted, indicative of the change in its chemical environment after reusing 4 times. Such blue shift might be due to the effect of Verway hopping (Mn^2+^ + Fe^3+^→Mn^3+^ + Fe^2+^) [41], which leads to the increase in electron cloud density around iron ions. Thus, both the emerged weak bands and the strengthening in the lattice absorption of Fe-O were not results of the change in material structure of the MnFe_2_O_4_/BGA composite.

#### 3.2.5. Possible Degradation Mechanism

**Figure 13:**
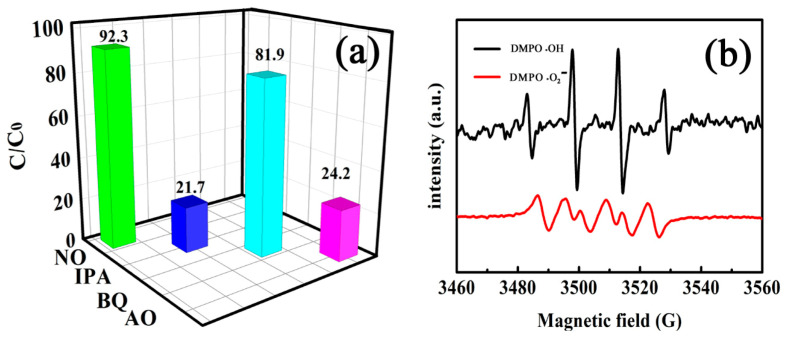
(**a**) Effect of radical scavengers on RhB degradation and (**b**) ESR spectra of DMPO-•OH and DMPO-•O_2_^−^ detected in the system of MnFe_2_O_4_/BGA-H_2_O_2_ under visible light.

It is well accepted that hydroxyl radical (•OH) is the specific active species in Fenton or Fenton-like systems. To identify the major reactive species formed in the present system, trapping experiments were performed with ammonium oxalate (AO), isopropanol (IPA), and benzoquinone (BQ) as the scavengers to quench h^+^, •OH, and •O_2_^−^, respectively. As shown in Figure 13a, the removal rate of RhB was decreased by 70.6% and 68.1% in the presence of IPA and OA, respectively, whereas the removal rate was only decreased 10.4% in the presence of BQ. Therefore, the photocatalytic degradation of RhB takes place mainly via photo-generated e^-^ and h^+^ on the MnFe_2_O_4_/BGA composite under visible light illumination.

On the other hand, the EPR spin-trap method, with 5,5-dimethy-1-pyrroline N-oxide (DMPO) as spin trapping active species, was performed to evaluate the radicals produced in the photodegradation of RhB under visible light illumination. As shown in Figure 13b, the characteristic quartets both for DMPO-•OH adducts with intensity ratios of 1:2:2:1 and for DMPO-•O_2_^−^ adducts with intensity ratios of 1:1:1:1 were clearly identified. Nonetheless, it should be noted that the relative concentration of •OH radicals is much higher than that of •O_2_^−^ radicals, because the intensity of DMPO-•OH adducts is about five times larger than that of DMPO-•O_2_^−^ adducts. Consequently, •OH and •O_2_^−^ radicals are proven to be the major and minor active species, respectively, in this visible light driven photocatalytic degradation of organic pollutants in the MnFe_2_O_4_/BGA/H_2_O_2_ photo-Fenton system.

The following electrochemistry and photoluminescence investigations strongly support that the MnFe_2_O_4_/BGA composite possesses higher electronic conductivity and electron transfer efficiency than MnFe_2_O_4_, BGA, and the MnFe_2_O_4_/GA composite. Hence, the photo-generated electrons and holes on the MnFe_2_O_4_/BGA composite are more effectively separated under visible light irradiation, which is mainly responsible for the excellent photocatalytic performance of the MnFe_2_O_4_/BGA composite in the degradation of organic pollutants under visible light irradiation. 

**Figure 14:**
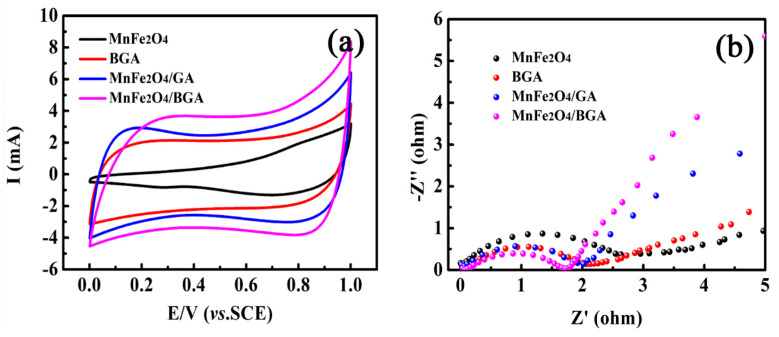
(**a**) CV curves and (**b**) EIS spectra of MnFe_2_O_4_, BGA, MnFe_2_O_4_/GA, and MnFe_2_O_4_/BGA.

It can be seen from Figure 14a that the MnFe_2_O_4_/BGA composite demonstrates the highest response current among the measured materials, indicative of its rapid electron transfer. The CV cures of BGA, MnFe_2_O_4_/GA, and MnFe_2_O_4_/BGA are of quasi-rectangular shapes, whereas that of MnFe_2_O_4_ is largely deviated from a quasi-rectangular shape. It should be noted that the loop area within the CV curve follows the sequence of MnFe_2_O_4_/BGA > MnFe_2_O_4_/GA > BGA > MnFe_2_O_4_, implying the highest electrochemical activity and electron transfer efficiency of MnFe_2_O_4_/BGA among these four materials, which is consistent with the sequence of their surface areas (see Figure 5). Moreover, as displayed in Figure 14b, the semicircle of the MnFe_2_O_4_/BGA composite in the high-frequency region is the smallest among the tested materials. Because a smaller semicircle in the electrochemical impedance spectroscopy (EIS) Nyquist plot means an overall smaller charge transfer resistance and a faster interfacial charge transfer, the electron mobility is greatly accelerated and the recombination rate of photo-generated electrons and holes is effectively inhibited when the MnFe_2_O_4_/BGA composite is applied as a visible light photocatalyst.

**Figure 15:**
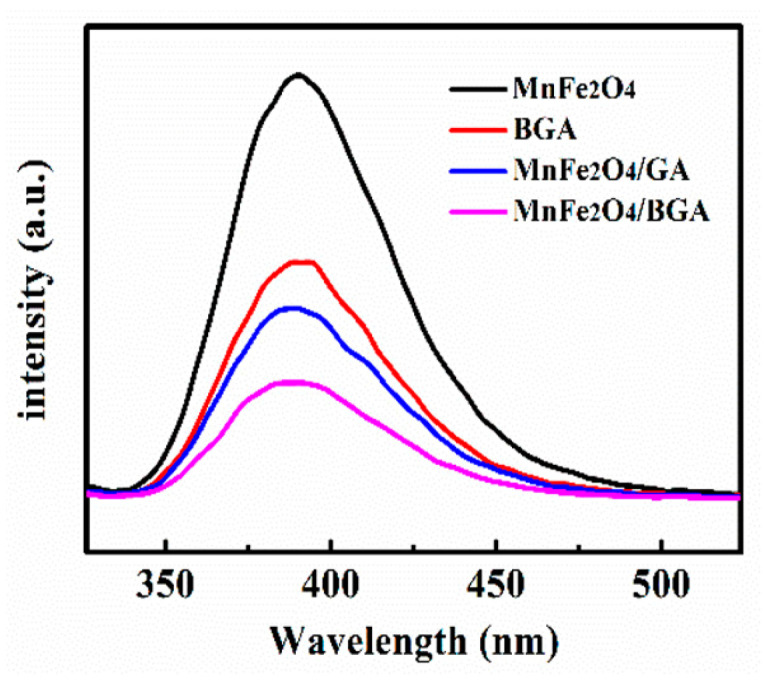
PL spectra of MnFe_2_O_4_, BGA, MnFe_2_O_4_/GA, and MnFe_2_O_4_/BGA.

It is well-known that the photoluminescence spectrum (PL) is an effective tool to estimate the recombination probability of photo-generated charge carriers [42]. As the emission signal is derived from the recombination of photo-induced e^−^, h^+^ pairs, the weaker the emission signal is, the higher the separation efficiency of charge carriers. From Figure 15, it can be seen that the emission signal trended lower and lower in the order of pure MnFe_2_O_4_, BGA, MnFe_2_O_4_/GA, and MnFe_2_O_4_/BGA, i.e., the recombination rate of the photo-induced e^−^, h^+^ pairs decreased in the same order. Therefore, the combination of BGA and MnFe_2_O_4_ can effectively inhibit the recombination of electron-hole pairs, which is mainly responsible for the excellent photo-Fenton performance of the MnFe_2_O_4_/BGA composite. It should be noted that the results extracted from Figure 15 are highly consistent with the photo-Fenton performance of pure MnFe_2_O_4_, BGA, MnFe_2_O_4_/GA, and MnFe_2_O_4_/BGA (Figure 8).

As a matter of fact, the catalytic degradation of organic pollutants involves their adsorption on photocatalysts and subsequent oxidation by reactive oxidation species (ROSs). Because pre-adsorption can generate high concentrations of organic pollutants around photocatalysts, the contact probability between organic pollutants and ROSs is naturally enhanced, which benefits the following photocatalytic degradation of organic pollutants. In contrast to pure MnFe_2_O_4_, the MnFe_2_O_4_ nanoparticles in MnFe_2_O_4_/BGA composite become smaller and are homogeneously loaded on the inner surface of BGA (Figure 1). Meanwhile, the 3D interconnected porous structure of the MnFe_2_O_4_/BGA composite provides numerous channels for the quick diffusion and adsorption of organic pollutant molecules, mainly due to the strong π-π bonding between 3D graphene aerogel and aromatic organic pollutants. Moreover, the introduction of boron atoms also brings some defects to the networks of reduced graphene oxide. The specific surface area of the MnFe_2_O_4_/BGA composite is much larger than those of BGA and MnFe_2_O_4_ (Figure 5), and its adsorption rate in the dark is much higher than those of BGA, MnFe_2_O_4_, and the MnFe_2_O_4_/GA composite (Figure 8a). Thus, it is concluded that many more organic pollutant molecules were absorbed in the MnFe_2_O_4_/BGA composite than in BGA, MnFe_2_O_4_, or the MnFe_2_O_4_/GA composite, because of its much larger number of active sites and the stronger organic pollutant adsorption on each active site.

Because of the semiconducting or semimetallic properties of such photocatalysts as MnFe_2_O_4_, MnFe_2_O_4_/GA, BGA, and MnFe_2_O_4_/BGA, light irradiation is indispensable for the photo-Fenton degradation of organic pollutants, and it is an important approach for the improvement in photo-Fenton performance to maximize the utilization efficiency of photocatalysts to visible light. On one hand, incident light reflectance and scattering multiplies in the 3D interconnected networks and channels (Figure 1c) of the MnFe_2_O_4_/BGA composite. On the other hand, the MnFe_2_O_4_/BGA composite exhibits a much higher absorption band than pure MnFe_2_O_4_ in the visible region (Figure 6a). Thus, the MnFe_2_O_4_/BGA composite has much larger visible light harvesting capability than pure MnFe_2_O_4_. This plays an important role in the enhanced degradation rate of organic pollutants on the MnFe_2_O_4_/BGA composite.

**Figure 16:**
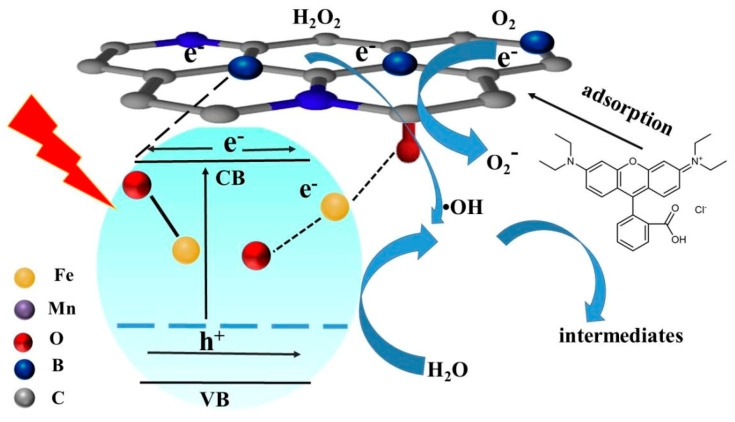
The mechanism for the photo-Fenton reaction by MnFe_2_O_4_/BGA.

More importantly, the combination of MnFe_2_O_4_ with BGA endows the MnFe_2_O_4_/BGA composite with much enhanced electron transfer efficiency and highly separated photo-excited electron-hole pairs, contributing largely to the improved photocatalytic organic pollutant degradation on the MnFe_2_O_4_/BGA composite under visible light irradiation. When a pure MnFe_2_O_4_ nanoparticle is irradiated by visible light (λ ≥ 420 nm), electrons and holes are generated in its valence band (VB) and conducting band (CB), respectively, owing to its semiconducting properties with an optical band gap of 2.09 eV (Figure 6b). In contrast, for the MnFe_2_O_4_/BGA composite, a new energy state is generated between the conduction and valence bands of MnFe_2_O_4_ originating from the combination of p-type BGA, and a narrow gap with an optical band gap of 1.75 eV (Figure 6b) between the new state and the conduction band is created. As shown in Figure 16, the electrons (e^−^) at the new state can be excited by visible light to the CB, leaving holes (h^+^) at the new state. The photo-excited electrons from the MnFe_2_O_4_ surface transfer rapidly to BGA because of the excellent electron transfer ability of BGA and the chemical bonding of Fe-O-B and Fe-O-C on the MnFe_2_O_4_/BGA interface. Because the electronegativity of C is larger than that of B, the sp^2^ structure of graphene is altered and more active sites are induced when boron is bonded with a carbon framework. Moreover, boron atoms have three valence electrons compared to four for carbon atoms. The introduction of boron atoms induces structural defects and uneven charge distributions in nearby sites, which can facilitate charge transfer between neighboring carbon atoms and further enhance electron transfer efficiency and electronic conductivity. FT-IR spectra (Figure 3d) evidenced the existence of B-C and O-B-O bonds in the MnFe_2_O_4_/BGA composite and the red shift in the lattice absorption of Fe-O in MnFe_2_O_4_. It is reasonable to speculate that there is a chemical bond between B doped in the sp^2^ structure of graphene and the oxygen on the surface of MnFe_2_O_4_ (B···O-Fe), and between the unreduced oxygen in graphene and the Fe on the surface of MnFe_2_O_4_ (C-O···Fe-O). Both of the chemical bonds act as the electron transfer channels from MnFe_2_O_4_ to BGA in the MnFe_2_O_4_/BGA composite. Moreover, because of the electron deficiency feature of the B element in the sp^2^ structure of graphene, BGAs are able to accumulate a large density of electrons. On one hand, these high-energy electrons (e^−^) are easily transported through the conjugated π-electron network of BGA and will react with the adsorbed H_2_O_2_ and O_2_ on the surface of BGA to form •OH and •O_2_^−^ radicals, respectively. On the other hand, the holes (h^+^) at the new state would react with H_2_O to yield extra •OH. Consequently, the absorbed organic pollutant molecules on the MnFe_2_O_4_/BGA composite can be directly oxidized to some stable intermediates largely by •OH radicals and partly by •O_2_^−^ radicals, and eventually to harmless mineral materials.

In the above-proposed possible photocatalytic degradation mechanism of the MnFe_2_O_4_/BGA composite, BGA actually plays the role of bridge between MnFe_2_O_4_ and H_2_O_2_ to accelerate the transfer of photo-excited electrons from the surface of MnFe_2_O_4_ particles to the adsorbed H_2_O_2_ and O_2_ to form •OH and •O_2_^−^ radicals. Thus, the recombination of photo-excited electrons and holes will be greatly depressed, leading to enhanced visible light photoactivity to the degradation of organic pollutants on the MnFe_2_O_4_/BGA composite. This is consistent with the above trapping results that confirmed the important roles of the •OH and •O_2_^−^ radicals.

## 4. Conclusions

The hydrothermally prepared MnFe_2_O_4_/BGA composite exhibited much enhanced photo-Fenton catalytic activity in the degradation of organic dyes owing to the synergistic effect between MnFe_2_O_4_ and BGA, which could be accounted for by the evenly distributed MnFe_2_O_4_ nanoparticles and the interconnected 3D porous network as well as the modulated electric charge distribution of BGA. Furthermore, because of the strong anchoring of MnFe_2_O_4_ nanoparticles on BGA, the MnFe_2_O_4_/BGA composite also demonstrated quite good stability for visible-light driven photocatalytic organic dye degradation. Moreover, it was found that the initial organic dye concentration, catalyst dosage, different content of MnFe_2_O_4,_ and H_2_O_2_ dosage could significantly affect organic dye photodegradation on the MnFe_2_O_4_/BGA composite.
